# Privacy-Preserving Verification of ML Preprocessing via Model Behavior Indicators

**DOI:** 10.1109/tp.2025.3628998

**Published:** 2025-11-04

**Authors:** WENBIAO LI, ANISA HALIMI, JAIDEEP VAIDYA, XIAOQIAN JIANG, ERMAN AYDAY

**Affiliations:** 1 Case Western Reserve University, Cleveland, OH 44106 USA; 2 IBM Research Europe, D15 HN66 Dublin, Ireland; 3 Rutgers University, Newark, NJ 07102-1811 USA; 4 UTHealth, Houston, TX 77030 USA

**Keywords:** Data preprocessing, differential privacy, explainable AI, local differential privacy, model auditing, tabular data

## Abstract

We present a privacy-preserving framework to verify whether a declared data preprocessing pipeline was correctly applied before training a machine learning model on sensitive data. The verifier has only black-box query access to the model and combines three behavior indicators: shift in prediction accuracy, Kullback-Leibler (KL) divergence between output distributions, and explanation vectors from Local Interpretable Model-agnostic Explanations (LIME) and SHapley Additive exPlanations (SHAP). The method requires neither the original training records nor ground-truth labels. It supports two tasks: (i) a binary decision on correctness and (ii) a multi-class diagnosis identifying which step is missing. Experiments on three tabular datasets (Diabetes, Adult-Income, Student-Record) show that the binary detector maintains over 75% F1 even under strong local differential privacy (ε=0.1). Machine-learning classifiers consistently outperform simple threshold rules in the binary setting, while the two approaches perform comparably for multi-class diagnosis. A label-free variant that clusters explanation vectors achieves competitive accuracy, enabling verification when no labeled pipelines are available. These results demonstrate a practical and scalable approach for safeguarding preprocessing integrity in privacy-sensitive machine learning workflows.

## I. INTRODUCTION

Machine learning (ML) has transformed domains ranging from health care to finance by enabling systems to learn directly from data [1], [2], [3], [4]. While algorithmic advances attract attention, the quality of the training data and, in particular, its preprocessing are equally decisive for model success.

Preprocessing converts raw records into a clean, model-ready table through tasks such as handling missing values, encoding categorical variables, normalizing numeric features, outlier removal, resampling, and duplicate elimination [5], [6], [7]. Neglecting a step can have subtle yet serious effects: unnormalized features with large magnitudes may dominate the learning objective, and duplicated rows can bias the model toward over-represented patterns. Such oversights often stem from varying domain expertise, heterogeneous toolchains, or miscommunication along a data pipeline.

Crucially, improper preprocessing does not always degrade common performance metrics. A model trained on flawed data can still achieve high test accuracy if the hold-out set is small or unrepresentative, masking problems that later surface in deployment. In high-stakes contexts such as health care or credit scoring, these hidden issues jeopardize safety and fairness.

Auditing a training pipeline is particularly hard when the underlying data are sensitive. Regulations or contracts may forbid downstream users from inspecting the raw records, leaving them with only black-box query access to the released model. Verification must therefore rely on privacy-preserving signals that reveal whether the declared preprocessing was truly applied-without exposing the data themselves.

### A. PROBLEM STATEMENT

We study six tabular preprocessing operations that are common in practice: (i) missing-value handling, (ii) categorical encoding, (iii) duplicate removal, (iv) outlier filtering, (v) feature normalizing, and (vi) resampling. Our goal is to decide, given only black-box queries to a model and a sample protected by local differential privacy (LDP) [8], whether the full pipeline was executed (binary verification) and, if not, to identify the omitted step (multi-class verification).

Traditional verification via accuracy or confusion-matrix metrics fails because those statistics are highly sensitive to the test-set distribution. Recent work on privacy-preserving audits focuses mainly on validating aggregate statistics [8]; extending such ideas to rich, non-linear ML behaviors is non-trivial.

### B. OUR APPROACH

We introduce a framework that fuses three model-behavior indicators:

- Accuracy shift between the released model and reference models,
- Kullback-Leibler (KL) divergence of output distributions, and
- Explanation vectors from Local Interpretable Model-agnostic…

Because these indicators capture complementary aspects of model behavior, their combination provides a robust signal of preprocessing integrity even when queries are perturbed by LDP noise (ε). A logistic classifier $f_{\theta }$ trained on synthetically generated reference models distinguishes compliant from non-compliant pipelines without access to the raw training data or their true labels.

### C. KEY INSIGHT

A model trained on an ε-LDP version of properly preprocessed data behaves more similarly to the released model than one trained after a step was skipped; this gap is detectable through the three indicators above.

### D. EMPIRICAL HIGHLIGHTS

On three real-world tabular datasets-CDC Diabetes, Adult Income, and Student Record-our verifier sustains ≥ 75% F1 in the binary task with strong privacy noise (ε=0.1). Machine-learning detectors outperform threshold baselines in the binary setting, while both approaches perform comparably in multi-class diagnosis. A label-free variant that clusters explanation vectors also achieves competitive accuracy, enabling verification when no labeled pipelines are available.

### E. CONTRIBUTIONS

1. A privacy-preserving framework that verifies tabular preprocessing integrity using a fusion of accuracy, KL divergence, and XAI-based explanations.
2. A black-box protocol that operates on an ε-LDP query set and requires neither the original training data nor their labels.
3. An evaluation demonstrating robustness across datasets, privacy budgets, and verification tasks.

The following short story previews our method; full details appear later in Fig. 3 and Section V.

## II. RELATED WORK

Verifying the integrity of machine-learning pipelines requires understanding (i) what preprocessing transformations are applied, (ii) how to audit models without direct data access, and (iii) which behavioral indicators reveal processing anomalies. This section reviews relevant work across data preprocessing methodologies, model auditing frameworks, and explainable AI foundations, then surveys privacy-preserving verification approaches and positions our contribution.

### A. DATA PREPROCESSING FOUNDATIONS

#### 1) PREPROCESSING TAXONOMIES AND SURVEYS

Data preprocessing encompasses diverse transformations that convert raw records into model-ready representations. Comprehensive surveys establish taxonomies covering discretization, normalization, feature extraction and selection, noise filtering, imbalanced data handling, and missing value imputation [9], [10]. These operations are fundamental to ML success yet prone to silent failures-undetected preprocessing errors can degrade deployed model performance even when validation metrics appear acceptable [11].

Recent work addresses preprocessing at scale. García et al. [9] analyze methods for distributed frameworks (Hadoop, Spark, Flink), revealing that preprocessing complexity grows nonlinearly with data volume and heterogeneity. Zhou et al. [12] introduce data-quality dimensions (accuracy, completeness, consistency) and survey 17 evaluation tools, showing that emerging techniques including LLMs can automate quality assessment but require verification mechanisms to ensure correctness.

#### 2) SPECIALIZED PREPROCESSING DOMAINS

Missing data handling remains a critical preprocessing challenge. Emmanuel et al. [13] provide a comprehensive survey of imputation approaches (KNN, Random Forest, SVM, ensembles) and missing-data mechanisms (MCAR, MAR, MNAR), demonstrating that imputation method choice significantly impacts downstream model behavior. Class imbalance presents similar challenges: Cândido et al. [14] systematically map 9,927 papers on sampling techniques across domains, proposing taxonomies for oversampling, undersampling, and hybrid approaches. Both missing-data imputation and classresampling techniques fundamentally alter model behavior patterns, making them essential targets for verification.

#### 3) AUTOMATED PREPROCESSING PIPELINES

The growing complexity of ML workflows has motivated automated data processing. Mumuni and Mumuni [15] survey automated preprocessing including data cleaning, labeling, missing-data imputation, categorical encoding, augmentation, and feature engineering, noting that AutoML methods optimize entire pipelines but introduce verification challenges-automated systems may apply transformations differently than declared. Kaur et al. [16] provide practical guidance on preprocessing issues (missing data, noise, inconsistency) and augmentation techniques, emphasizing that understanding transformation effects on model behavior is essential for verification.

#### 4) GAP IN PREPROCESSING VERIFICATION

While extensive literature documents preprocessing techniques and their impacts, minimal research addresses verifying that declared preprocessing was correctly applied, especially under privacy constraints where raw data cannot be inspected. This gap motivates our behavioral verification approach.

### B. MODEL AUDITING AND TESTING FRAMEWORKS

#### 1) SYSTEMATIC ML TESTING

Testing ML systems differs fundamentally from traditional software testing due to the oracle problem-lack of ground-truth labels for many test cases. DeepXplore [17] addresses this through differential testing, introducing neuron coverage metrics and using multiple models as cross-referencing oracles. The insight that models exhibit unexpected differential behaviors under incorrect preprocessing directly informs our verification approach. Metamorphic testing provides complementary techniques: Xie et al. [18] demonstrate that defining metamorphic relations-properties classifiers should satisfy under input transformations-enables validation without ground truth, which is crucial for black-box preprocessing verification.

Riccio et al. [19] provide a systematic mapping of 70 ML testing papers, classifying approaches by test adequacy criteria, input generation algorithms, and oracle types. This taxonomy reveals that most testing focuses on model outputs rather than pipeline integrity, and existing test oracles (e.g., prediction consistency, fairness metrics) do not directly address preprocessing correctness. Our work extends this landscape by introducing behavior-indicator-based oracles specifically designed for preprocessing verification.

#### 2) ML SYSTEM ENGINEERING CHALLENGES

Software engineering research has identified unique challenges in ML systems. Amershi et al. [20] document a nine-stage ML workflow through empirical study of Microsoft teams, revealing that discovering, managing, and versioning data is fundamentally more complex than traditional software engineering. Data versioning failures and model entanglement-where seemingly independent components develop hidden dependencies-create cascading effects when preprocessing changes, requiring systematic auditing. Sculley et al. [21] formalize ML-specific technical debt including boundary erosion, hidden feedback loops, and configuration complexity, arguing that system-level interactions incur massive maintenance costs that code-level testing cannot address.

#### 3) ML PIPELINE VALIDATION AND REPRODUCIBILITY

Complementary frameworks address pipeline verification from reproducibility perspectives. Zheng and Stodden [22] introduce the Idealized Machine Learning Pipeline (IMLP), a conceptual model emphasizing verification of each component from raw data through preprocessing to model estimation. Kaminwar et al. [23] develop structured verification methods for industrial ML, including unit tests to detect changes in preprocessing workflows. These reproducibility-focused approaches assume access to pipeline execution details; in contrast, our framework operates on black-box models with only query access-a critical distinction for privacy-sensitive or adversarial settings.

#### 4) PROVENANCE TRACKING AND METADATA

Automated provenance tracking provides complementary verification mechanisms. Schelter et al. [24] present a lightweight system for extracting, storing, and managing ML metadata (hyperparameters, dataset schemas, model architectures), enabling auditors to verify preprocessing integrity through metadata analysis without accessing sensitive data. Schlegel and Sattler [25], [26] extend this with W3C-PROV-compliant provenance graphs (MLflow2PROV) capturing Git and MLflow activities, supporting querying and analysis of transformation lineages. While provenance systems track what was executed, our behavioral verification validates whether declared preprocessing was correctly applied-addressing the gap when execution logs may be unavailable, unreliable, or deliberately falsified.

#### 5) BLACK-BOX TESTING APPROACHES

Recent work explores testing ML systems through API-level interactions. Wan et al. [27] introduce Keeper, which designs pseudo-inverse functions that empirically reverse ML tasks, incorporating them into symbolic execution to generate relevant test inputs. The pseudo-inverse concept suggests preprocessing verification strategies: checking whether transformations maintain expected inverse relationships through observable model behavior. Prinster et al. [28] audit predictive uncertainty under covariate shift, relevant for detecting preprocessing-induced distribution changes. Huang et al. [29] verify training data composition through black-box queries. These works audit model properties or data usage; we specifically target preprocessing integrity through behavior indicators that capture preprocessing-specific model characteristics.

### C. EXPLAINABLE AI FOUNDATIONS

#### 1) EXPLANATION METHODS WITH FORMAL FOUNDATIONS

Explainable AI (XAI) provides behavioral indicators for our verification framework. Lundberg and Lee [30] introduce SHAP (SHapley Additive exPlanations), a unified framework assigning feature importance values based on game-theoretic Shapley values with provable uniqueness and desirable properties (local accuracy, missingness, consistency). SHAP unifies six prior methods including LIME and DeepLIFT. Ribeiro et al. [31] propose LIME (Local Interpretable Model-agnostic Explanations), which explains predictions by learning interpretable models locally around individual predictions in a model-agnostic manner, working across text, image, and tabular data. For deep networks, Sundararajan et al. [32] present Integrated Gradients with axiomatic foundations (Sensitivity, Implementation Invariance), computing integrals of gradients along paths from baseline to input.

**FIGURE 1.**
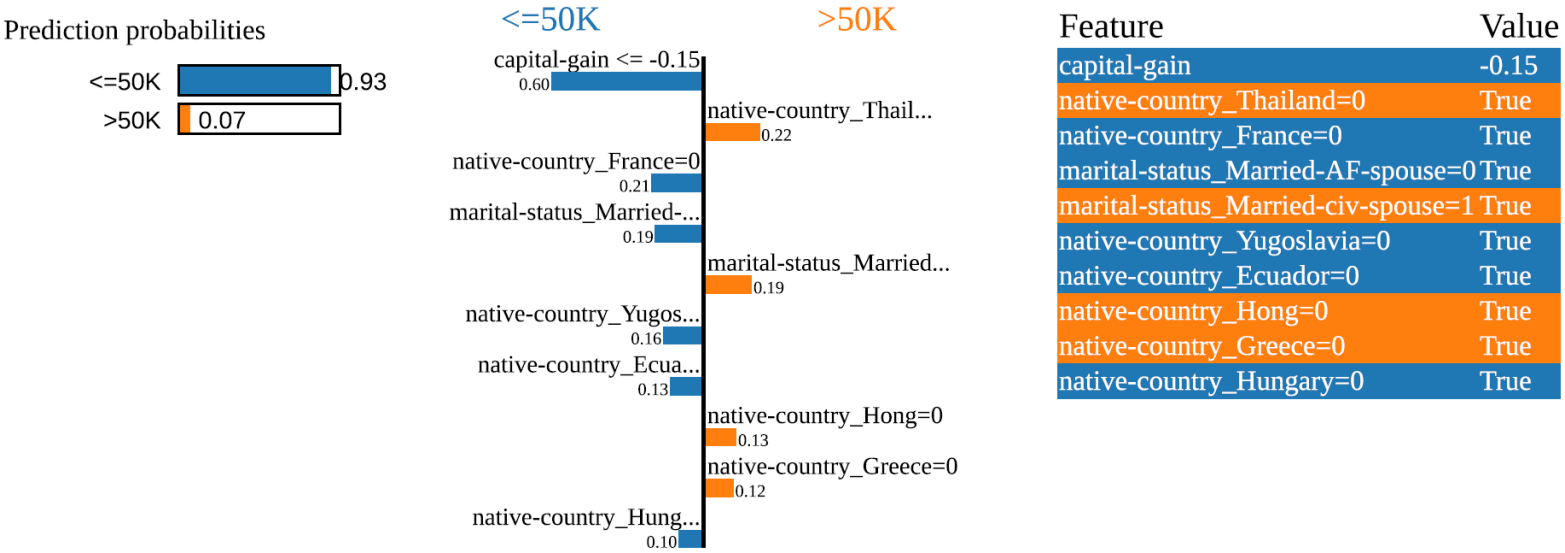
Example LIME explanation for an Adult Income instance.

**FIGURE 2.**
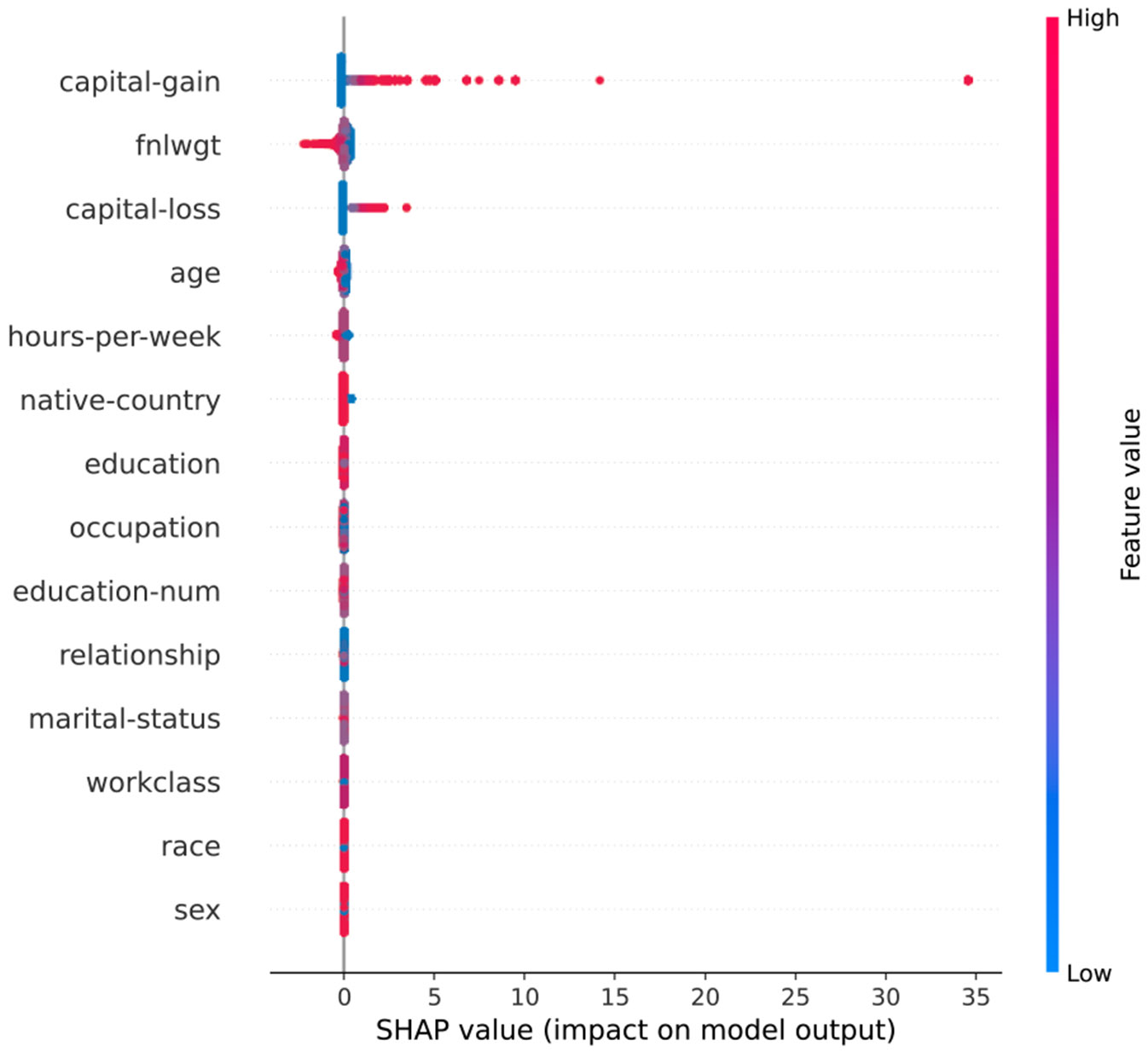
Corresponding SHAP values for the same instance.

These explanation methods are directly applicable to preprocessing verification: SHAP quantifies how preprocessing transformations affect feature contributions to predictions; LIME’s model-agnostic approach enables verification across different architectures; and Integrated Gradients reveals how preprocessing effects propagate through deep networks. Our framework leverages SHAP and LIME (Fig. 1 and 2) as discriminative behavioral indicators because they capture model decision-making processes that preprocessing manipulations fundamentally alter.

#### 2) INTERPRETABILITY THEORY AND EVALUATION

Establishing when explanations reliably indicate model behavior requires theoretical grounding. Doshi-Velez and Kim [33] provide rigorous definitions distinguishing interpretability from explainability, proposing evaluation approaches (application-grounded, human-grounded, functionally-grounded) and discussing when interpretability is necessary. This framework guides determining which explanation types are most appropriate for preprocessing verification-we require functionally-grounded evaluation showing explanations discriminate between correct and incorrect preprocessing. Rudin et al. [34] survey fundamental principles and identify ten technical challenges in interpretable ML, emphasizing inherently interpretable models over post-hoc explanations for high-stakes decisions. While we use black-box models, the principles establish that model behavior can be made transparent through explanations, validating our approach. Rudin [35] argues that for critical applications, inherently interpretable models achieve comparable accuracy to black boxes while providing genuine transparency, supporting the use of behavioral indicators as verification mechanisms.

#### 3) XAI IN AUDITING CONTEXTS

Practical applications demonstrate XAI’s utility for verification. Zhang et al. [36] introduce XAI techniques to auditing practitioners, showing how LIME and SHAP meet audit documentation and evidence standards for assessing risk of material misstatement. The audit framework parallels preprocessing verification needs: both require demonstrable evidence that systems work correctly and outputs can be trusted. For privacy-preserving contexts, Nguyen et al. [37] provide the first comprehensive survey on privacy-preserving model explanations, analyzing privacy attacks on XAI methods (membership inference, model extraction, data reconstruction) and cataloging defense mechanisms. This work is critical because verification often requires sharing explanations across organizational boundaries-understanding privacy implications ensures that behavioral indicators enable transparent verification while protecting sensitive training data and proprietary preprocessing techniques.

### D. PRIVACY-PRESERVING VERIFICATION IN PRACTICE

#### 1) BIOMETRIC VERIFICATION

Secure face matching with nearest-neighbor protocols on edge devices [38] and speaker verification via secure multiparty computation [39] protect sensitive features, but their cryptographic workloads scale poorly and are tightly coupled to biometric data formats.

#### 2) MODEL-SPECIFIC FRAMEWORKS

pvCNN [40] enables privacy-preserving testing for convolutional networks, and other schemes secure data integrity in mobile edge computing [41] or smart-grid control. These solutions are bound to particular architectures or infrastructures and therefore do not generalize to heterogeneous, black-box models.

#### 3) MODEL-BEHAVIOR COMPARISON

Zest [42] compares ML models by computing cosine similarity between Local Interpretable Model-agnostic Explanations (LIME) vectors, supporting tasks such as model reuse detection and machine unlearning. Its focus, however, is model similarity rather than training-data correctness-a gap our framework addresses by using explanation vectors specifically to verify preprocessing integrity.

### E. POSITIONING OF PRESENT WORK

Our framework synthesizes insights from preprocessing methodologies, model auditing, and explainable AI to address a gap at their intersection: verifying declared preprocessing was correctly applied under strict privacy constraints. Unlike preprocessing surveys that document transformation techniques [9], [13], we provide mechanisms to audit their correct application. Unlike model testing frameworks that focus on output correctness [17], [18], we target pipeline integrity upstream of final predictions. Unlike pipeline validation tools that require execution access [22], [23] or provenance tracking that assumes honest reporting [25], we verify through behavioral indicators that detect discrepancies between declared and actual preprocessing. Unlike XAI methods that explain individual predictions [30], [31], we use explanations as discriminative indicators of preprocessing state.

Specifically, we combine three complementary behavior indicators: (i) accuracy shift quantifying prediction quality differences, (ii) Kullback–Leibler divergence measuring output distribution changes, and (iii) LIME and SHAP explanation vectors capturing feature-attribution patterns. Verification operates on ε-locally differentially private queries, requiring no access to raw training data, internal model parameters, or ground-truth labels. The approach is architecture-agnostic, supports strict privacy budgets (ε≥0.1), and targets the entire training pipeline’s integrity rather than the final model alone.

While prior work addresses related challenges in biometric verification [38], model-specific testing [40], model comparison [42], pipeline reproducibility [22], provenance tracking [25], and black-box auditing [28], [29], to our knowledge this is the first framework systematically addressing preprocessing verification through privacy-preserving behavioral indicators. This fills a critical gap as ML systems increasingly process sensitive data under regulatory constraints (HIPAA, GDPR) that prohibit direct inspection while requiring verifiable integrity guarantees.

## III. BACKGROUND

This section reviews the key concepts used in the proposed framework: differential privacy, Local Interpretable Model-agnostic Explanations (LIME), SHapley Additive exPlanations (SHAP), Kullback-Leibler (KL) divergence, and tabular-data preprocessing.

### A. DIFFERENTIAL PRIVACY

Differential privacy (DP) provides a formal guarantee that the output of an algorithm changes only marginally when a single record in its input dataset is modified [43]. The privacy budget ε quantifies this stability: smaller values yield stronger privacy at the expense of utility.

Local differential privacy (LDP) removes the need for a trusted curator by perturbing each record at the source [44]. A common mechanism adds Laplace noise,

(1) F(x)=f(x)+Lap(s∕ε),

where f is a numeric query with $\ell_{1}$-sensitivity s and Lap(λ) is a Laplace random variable with scale λ. Its probability density (centered at zero) is $\frac{1}{2\lambda }\;\text{exp}(-|t|∕\lambda )$ for real t. In our workflow the researcher adds Laplace noise to the training set before sharing it; the verifier receives only this LDP-protected data and the released model.

### B. LOCAL INTERPRETABLE MODEL-AGNOSTIC EXPLANATIONS

LIME approximates the local behavior of a black-box model f at an instance x by fitting a sparse linear surrogate g:

(2) $$\underset{g\in 𝒢}{\text{min}}\;ℒ(f,g,\pi_{x})+\Omega (g),$$

where $\pi_{x}$ is an exponential kernel that weights perturbed samples and Ω(⋅) enforces sparsity [45]. The coefficients of g constitute an explanation vector. Distances between such vectors-computed for identical queries under different models-serve as an indicator of behavioral similarity.

### C. SHAPLEY ADDITIVE EXPLANATIONS

SHAP assigns an additive contribution $\phi_{i}$ to each feature i such that

(3) $$f(x)=f_{0}+\sum_{i=1}^{N}\phi_{i}(f,x),$$

where $f_{0}$ is the dataset expectation [30]. Shapley values satisfy local accuracy and consistency, making them model-agnostic and comparable across architectures. The verifier therefore treats SHAP vectors exactly as LIME vectors when forming its feature set.

### D. KULLBACK-LEIBLER DIVERGENCE

For discrete distributions P and Q, the KL divergence is

(4) $$D_{\text{KL}}(P\;∥\;Q)=\sum_{i}P(i)\;\log\;\frac{P(i)}{Q(i)},$$

and equals zero if and only if P=Q [46]. Computing $D_{\text{KL}}$ between the predictive distributions of a reference model (correct pipeline) and a target model (suspect pipeline) yields a scalar indicator of behavioral drift.

### E. PREPROCESSING IN TABULAR MACHINE LEARNING

Two steps are indispensable for tabular data: missing-value handling and categorical encoding. Four further operations are often applied conditionally: duplicate removal, outlier filtering, feature scaling, and class-imbalance resampling [7]. The present study follows the canonical order recommended by scikit-learn and later evaluates omissions of each optional step (Section VI). Behavior indicators derived from reference and target models enable the verifier to detect which step, if any, was skipped.

## IV. SYSTEM, THREAT, AND PRIVACY MODELS

**FIGURE 3.**
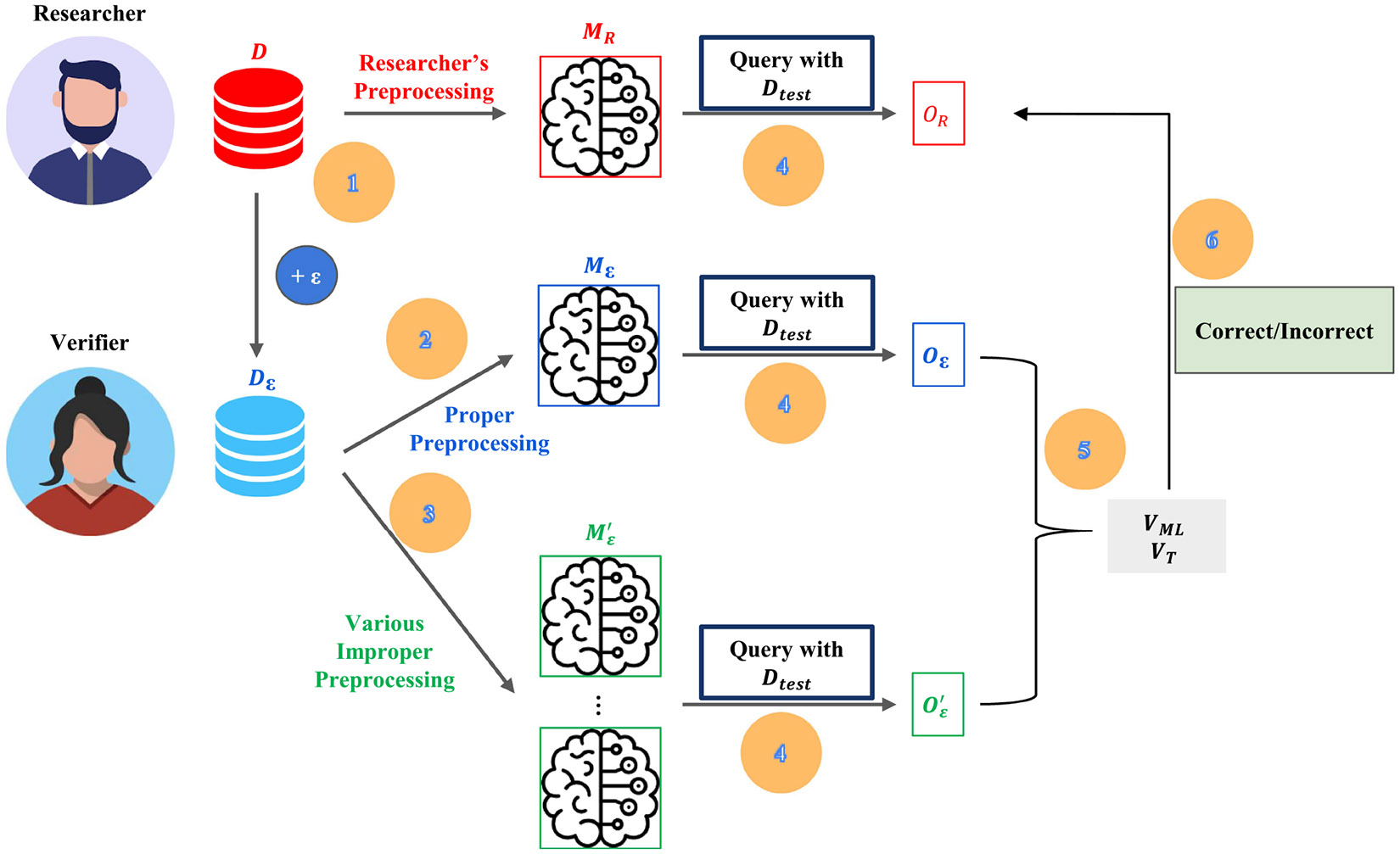
Overview of the verification workflow. The researcher publishes a black-box model and an LDP dataset $D_{\varepsilon }$. The verifier trains reference models under proper and improper pipelines, queries all models on a shared test set, collects behavior indicators, and decides correctness via machine-learning ($V_{\mathrm{ML}}$) or threshold rules ($V_{T}$).

Two entities participate in the framework (Fig. 3): *(i) the researcher*, who trains and releases a model together with a locally differentially private dataset, and *(ii) the verifier*, who audits whether the declared preprocessing pipeline was followed.

### A. SYSTEM MODEL

The researcher discloses the model architecture (e.g., logistic regression) but not its parameters, and releases an LDP dataset $D_{\varepsilon }$ for auditing. The verifier issues black-box queries drawn from its own cohort $D_{\text{test}}$ and, for each sample, collects the behavior tuple $O=(E,A,D_{\text{KL}})$. Reference models $M_{\varepsilon }$ and $M_{\varepsilon }^{\prime }$ are trained on $D_{\varepsilon }$ and synthetically corrupted variants, respectively; their behavior indicators are compared with those of $M_{R}$.

The following example illustrates how the symbols in Table 1 operate in practice; Section V formalizes the full workflow.

### B. THREAT MODEL

#### Researcher:

The researcher is honest but fallible: preprocessing mistakes may occur inadvertently. Deliberate data poisoning is out of scope because it cannot be detected without the raw data.

#### Verifier:

The verifier may attempt to extract private information from $D_{\varepsilon }$. Among standard threats-membership-, attribute-, and re-identification attacks-membership inference is most relevant, because all attributes are already revealed in noisy form. Prior work shows that differential privacy mitigates membership inference effectively [47], [48]. Here, the researcher’s data are protected by LDP, and the verifier’s own queries are likewise perturbed before they reach $M_{R}$:

##### 1) WHY THE VERIFIER APPLIES ε-LDP TO ITS QUERIES

Queries originate from a private cohort (e.g., clinical records). A malicious model owner could log repeated calls and correlate them with auxiliary knowledge to launch membership or attribute inference [48], [49]. Each query in $D_{\text{test}}$ is therefore perturbed with Laplace noise, producing an LDP set that bounds the adversary’s posterior gain. Section VI shows that the resulting utility loss remains modest for ε≤1, confirming that verifier-side protection is both practical and necessary.

## V. PROPOSED FRAMEWORK

The framework (Table. 2 and Fig. 3) determines whether the released model was trained with the declared preprocessing pipeline, without access to the model parameters or the raw training data.

### A. RESEARCHER PHASE (STEP 1IN FIG. 3)

The researcher applies the declared pipeline to a private dataset D, trains a model $M_{R}$, and releases the architecture (e.g., logistic regression) but not the parameters, and an LDP-protected dataset $D_{\varepsilon }$ generated with the Laplace mechanism (Section III-A).

Each feature value x is perturbed by Lap(0,s∕ε), where s is the $l_{1}$-sensitivity. Optional clipping keeps the noisy values within the original domain.

When queried with $D_{\text{test}}$, the model returns an explanation vector $E_{R}$ (LIME or SHAP). Predicted labels ${\hat{y}}_{R}$ are not required.

### B. VERIFIER PHASE (STEPS 2–6)

#### 1) STEP 2: REFERENCE-MODEL CONSTRUCTION

Using $D_{\varepsilon }$ and the released architecture, the verifier trains $M_{\varepsilon }$ under the proper pipeline, and a set of models $M_{\varepsilon }^{\prime }$ obtained by omitting exactly one step (details in Section VI).

#### 2) STEPS 3–4: QUERYING AND INDICATOR EXTRACTION

For each model and each sample in $D_{\text{test}}$, the verifier collects

$$O=[E,\;A,\;D_{\text{KL}}],$$

where E is the explanation vector, A the accuracy on $D_{\text{test}}$, and $D_{\text{KL}}$ the KL divergence between the model’s output distribution and that of $M_{\varepsilon }$.

**Table:** 

Algorithm 1 Privacy-Preserving Verifier for Preprocessing Integrity.
$$\begin{array}{cc} \; & \;\mathrm{Require}:\text{Released model}\;M_{R};\;\text{declared pipeline}\;𝒫;\;\text{test set}\\ \; & \;D_{\text{test}};\;\text{privacy budget}\;\varepsilon \\ \; & \;\mathrm{Ensure}:\text{Predicted missing-setp set}\;\hat{S}\subseteq 𝒫\\ \; & \;1:D_{\varepsilon }\leftarrow \text{LAPLACEDP}(D_{\text{test}},\varepsilon )\;▷\;\text{apply}\;\varepsilon \text{-LDP to}\\ \; & \;2:\mathrm{for}\;\mathrm{all}\;S\subseteq 𝒫\;\mathrm{with}\;S\neq \emptyset \;\mathrm{do}\\ \; & \;3:\;\text{Train reference model}\;M_{S}\;\text{on}\;D_{\varepsilon }\;\text{using pipeline}\\ \; & \;{𝒫}_{S}\\ \; & \;4:\;\phi_{S}\leftarrow \\ \; & \;[\Delta A(M_{S},M_{R})\;D_{\text{KL}}(M_{S},M_{R}),\;\text{ExpDist}(M_{S},M_{R})]\\ \; & \;5:\mathrm{end}\;\mathrm{for}\\ \; & \;6:\hat{S}\leftarrow {\text{arg max}}_{S}f_{\theta }(\phi_{S})\;▷\;f_{\theta }:\text{logistic detector}\\ \; & \;7:\mathrm{return}\;\hat{S} \end{array}$$

#### 3) STEPS 5–6: DECISION RULES

##### 3) Machine-learning detector:

A training set is built from $\langle O_{\varepsilon },0\rangle$ and $\langle O_{\varepsilon }^{\prime },1\rangle$ with either binary or multi-class labels. The classifier $V_{\text{ML}}$ predicts the label of $O_{R}$.

##### 3) Threshold detector:

Cosine distances between $O_{\varepsilon }$ and each $O_{\varepsilon }^{\prime }$ are averaged to form a threshold τ. If the distance between $O_{R}$ and $O_{\varepsilon }$ exceeds τ, the pipeline is flagged as incorrect.

### C. VERIFYING ALGORITHM

#### Complexity:

In the Algorithm 1, at most $2^{\sigma }-1$ reference models are trained (15 when σ=4); runtime is $O(2^{\sigma }T_{\text{train}})$ and memory $O(2^{\sigma }d)$, where d is the feature dimension.

#### Variable-Order Extension:

Data pipelines sometimes reorder independent steps. Let $\left\{{𝒫}^{\left(1\right)},\ldots ,{𝒫}^{\left(k\right)}\right\}$ be k≪σ! plausible orders (often k≤6). Running Algorithm 1 under each order and merging the predicted sets into ${\hat{S}}_{\text{union}}={⋃}_{i=1}^{k}{\hat{S}}^{\left(i\right)}$ yields an order-agnostic decision with at most k-fold cost. Pilot tests on the Adult dataset showed no measurable drop in F1 compared with the canonical order.

## VI. EVALUATION

We evaluate the framework on three public datasets under a variety of improper preprocessing scenarios. We measure both verification effectiveness and privacy leakage.

### A. DATASETS

We use three public, person-level datasets spanning health care, census, and education. Table 3 summarizes their characteristics.

#### 1) CDC DIABETES HEALTH INDICATORS

Published by the U.S. Centers for Disease Control and Prevention and mirrored on Kaggle,^1^ the file contains 253,680 survey responses with 21 attributes (14 categorical, 7 numeric). The positive class (diagnosed diabetes) accounts for 35,346 records (13.9% ). All variables are de-identified and aggregated.

#### 2) ADULT INCOME

The UCI census dataset [50] has 48,842 records, 14 features (8 categorical, 6 numeric), and a binary label indicating income > $50 k. The minority (high-income) class covers 24.0% of instances.

#### 3) STUDENT RECORD

The “Predict Students’ Drop-out and Academic Success” corpus [51] contains 4,424 undergraduate trajectories with 36 predictors (25 categorical, 11 numeric) and a three-class target (drop-out / enroll / graduate). Classes are skewed: drop-out 22.7%, enroll 38.5%, graduate 38.8% .

### B. IMPROPER PREPROCESSING SCENARIOS

The canonical pipeline applies two required steps-dropping missing values and categorical encoding-followed by four optional steps (drop duplicates, remove outliers, scale features, resample classes) in that order. Improper variants omit one or more optional steps from the end while respecting order, yielding 14 distinct pipelines used to train erroneous reference models $M_{\varepsilon }^{\prime }$.

### C. PRIVACY ANALYSIS

**FIGURE 4.**
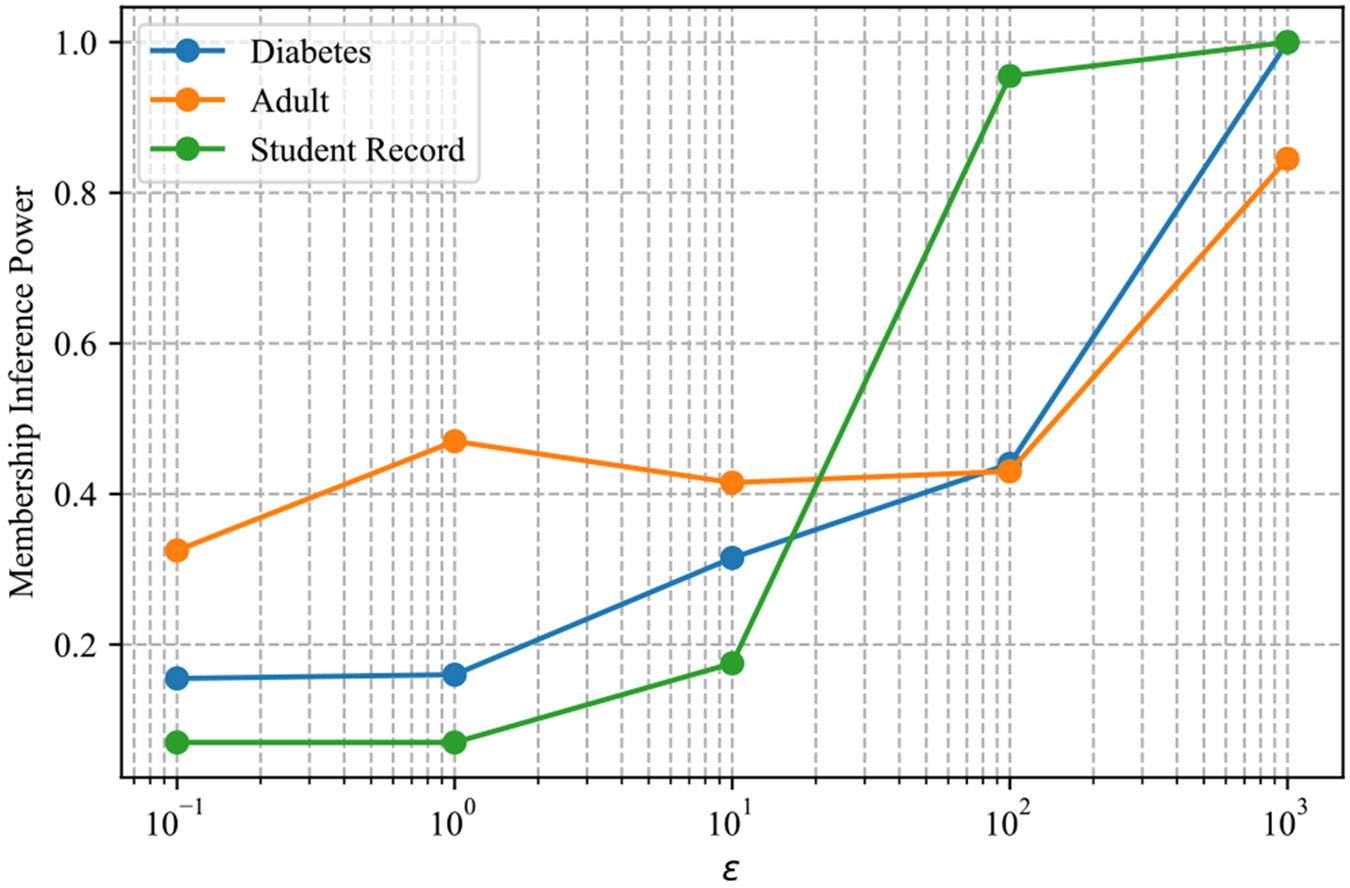
Membership-inference attack accuracy (higher is worse) versus privacy budget ε (log scale).

We estimate membership-inference (MI) power via a Hamming-distance attack. For a 5% false-positive rate, we derive a distance threshold γ on a control (non-member) set and report attack accuracy on a case (member) set. Fig. 4 shows that attack power increases monotonically with the privacy budget ε∈{0.1,1,10,100,1000}, as expected. The Student Record data are most vulnerable; Diabetes and Adult remain at or below 0.5 attack accuracy for ε≤100. Sharing LIME or SHAP explanations increases MI accuracy by at most 0.04, consistent with Shokri et al. [52].

#### Proposition 1 (Per-record LDP guarantee):

Let $D_{\text{test}}$ be any multiset of query records and let ℳ be the mechanism that (i) perturbs each record $x\in D_{\text{test}}$ by independent Laplace noise with scale s∕ε (after clipping so that the $l_{1}$-sensitivity s is bounded), and (ii) feeds the resulting $D_{\varepsilon }$ to Algorithm 1. Then for every record $x\in D_{\text{test}}$ and every pair of neighboring datasets D, $D^{\prime }$ that differ only in x,

$$\text{Pr}[ℳ(D)=o]\leq e^{\varepsilon }\;\text{Pr}[ℳ(D^{\prime })=o].$$

For all measurable outputs o. Hence ℳ satisfies ε-local differential privacy for each record.

#### Sketch of proof:

The identity query f(x)=x on a single, clipped record has bounded $l_{1}$-sensitivity s. The Laplace mechanism with scale s∕ε therefore provides ε-DP for each record in $D_{\text{test}}$ [43]. Algorithm 1 is pure post-processing of $D_{\varepsilon }$, which cannot weaken privacy by the post-processing theorem.

### D. EXPERIMENTAL SETUP

Each dataset is split 80/20 into train and test folds; 500 test records form $D_{\text{test}}$. Logistic regression is the default released-model architecture; decision tree and random forest variants are included for robustness. All experiments are repeated five times with different random seeds; we report means. Privacy budgets follow ε∈{0.1,1,10,100,1000,∞}. Verification is evaluated in both binary (“any step omitted?”) and multi-class (“which step omitted?”) settings. We report verification accuracy for both binary and multi-class tasks; random baselines are 0.5 and 1/15, respectively (one “proper” plus 14 improper cases).

The verifier distinguishes between 15 preprocessing configurations (cases 0–14), systematically covering all possible combinations of the 4 optional steps. Table 4 enumerates these cases: case 0 applies all steps (proper preprocessing), cases 1–4 omit one step each (𝒞(4,1)=4 combinations), cases 5–10 omit two steps simultaneously (𝒞(4,2)=6 combinations), and cases 11–14 omit three steps (𝒞(4,3)=4 combinations). This design enables evaluation of verification accuracy when the researcher omits multiple preprocessing operations, addressing the question of whether behavior indicators remain discriminative when model deviation is large due to compounded omissions.

### E. VERIFICATION RESULTS

We evaluate the framework on Diabetes, Adult Income, and Student Record using two explanation methods (LIME and SHAP) and three released-model architectures (logistic regression, random forest, decision tree). Two verifiers are compared: (i) an ML-based detector (logistic regression on the fused indicator vector: explanations, accuracy shift, and $D_{\text{KL}}$), and (ii) a threshold-based rule that uses a cosine-distance threshold over the same indicators. Each training pipeline is encoded as a case ID reflecting its combination of preprocessing steps; there are 15 case IDs in total. We report verification accuracy for both binary and multi-class tasks; random baselines are 0.5 and 1/15, respectively (one “proper” plus 14 improper cases).

#### 1) BINARY VERIFICATION WITH THE ML-BASED DETECTOR

In the binary setting (proper vs. improper), the ML-based verifier achieves consistently high accuracy across architectures, explainers, and datasets- typically > 0.90 for ε≥10, often approaching 0.95 on *Diabetes*. Trends vary little with ε, indicating that the combined indicators remain discriminative even under stronger privacy noise.

#### 2) MULTI-CLASS VERIFICATION WITH THE ML-BASED DETECTOR

**FIGURE 5.**
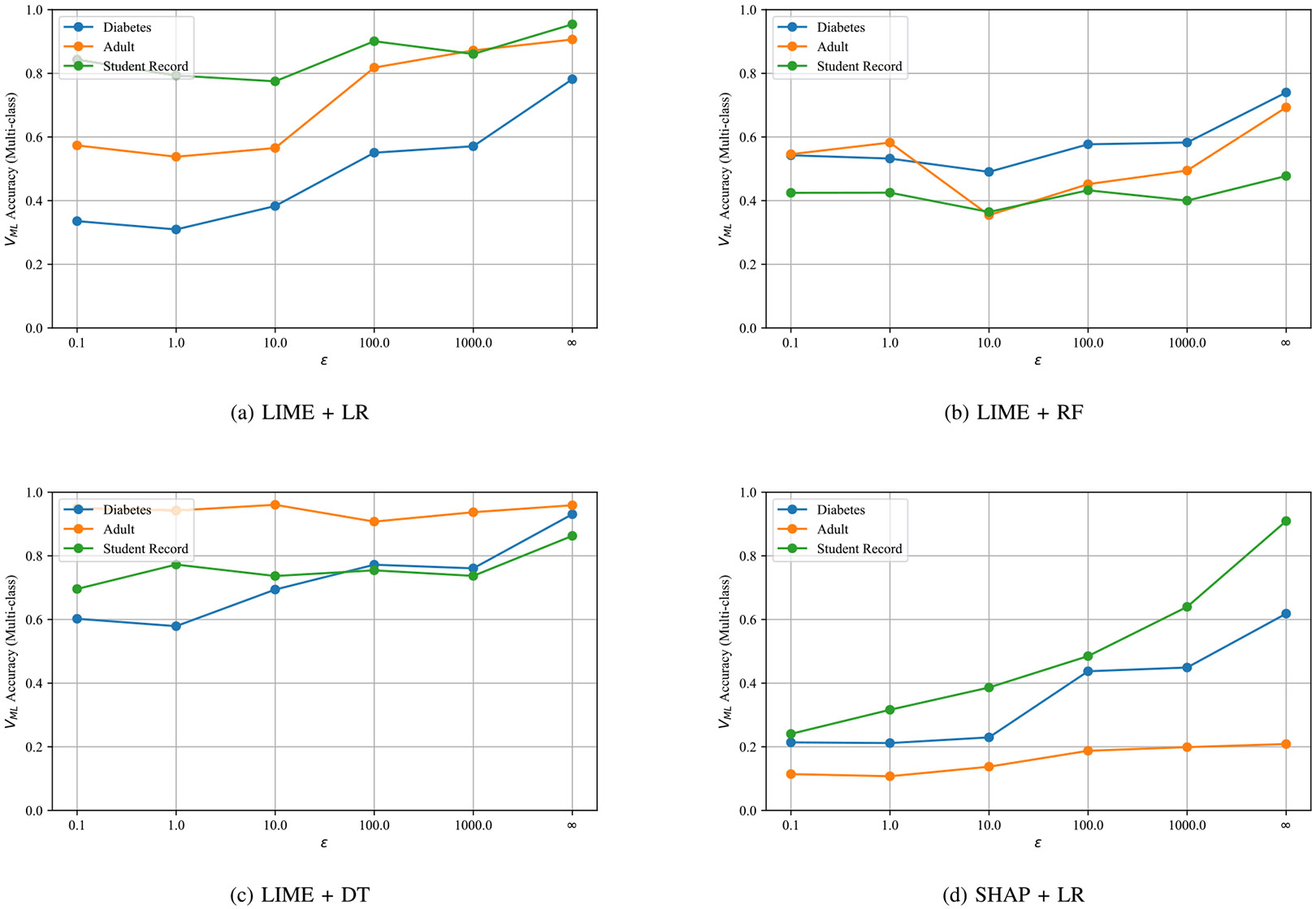
Multi-class verification accuracy across architectures, explainers, and privacy budgets.

We next consider the multi-class task (identify the missing step) across ε∈{0.1,1,10,100,1000,∞} (Fig. 5). Accuracy improves with larger ε (less noise), as expected. With LIME + logistic regression, *Diabetes* rises from 0.70 at ε=0.1 to 0.85 at ε=∞, while *Adult* and *Student* reach ≈ 0.78 and 0.81. LIME + decision tree and LIME + random forest are slightly lower overall but follow similar trajectories. SHAP + logistic regression performs best, with *Diabetes* reaching 0.88 and the other two datasets exceeding 0.80 at high ε. Differences across architectures and explainers are modest (typically within 0.05-0.08), suggesting robustness of the approach.

#### 3) BINARY VERIFICATION WITH THE THRESHOLD RULE

The threshold-based verifier also performs strongly on the binary task. Across all combinations, accuracy generally exceeds 0.90 even at ε=0.1; for example, SHAP + logistic regression on *Diabetes* is > 0.95, and *Adult* and *Student* remain > 0.92. This confirms that the binary decision is comparatively easy and that a lightweight rule can suffice when interpretability or simplicity is preferred.

#### 4) MULTI-CLASS VERIFICATION WITH THE THRESHOLD RULE

**FIGURE 6.**
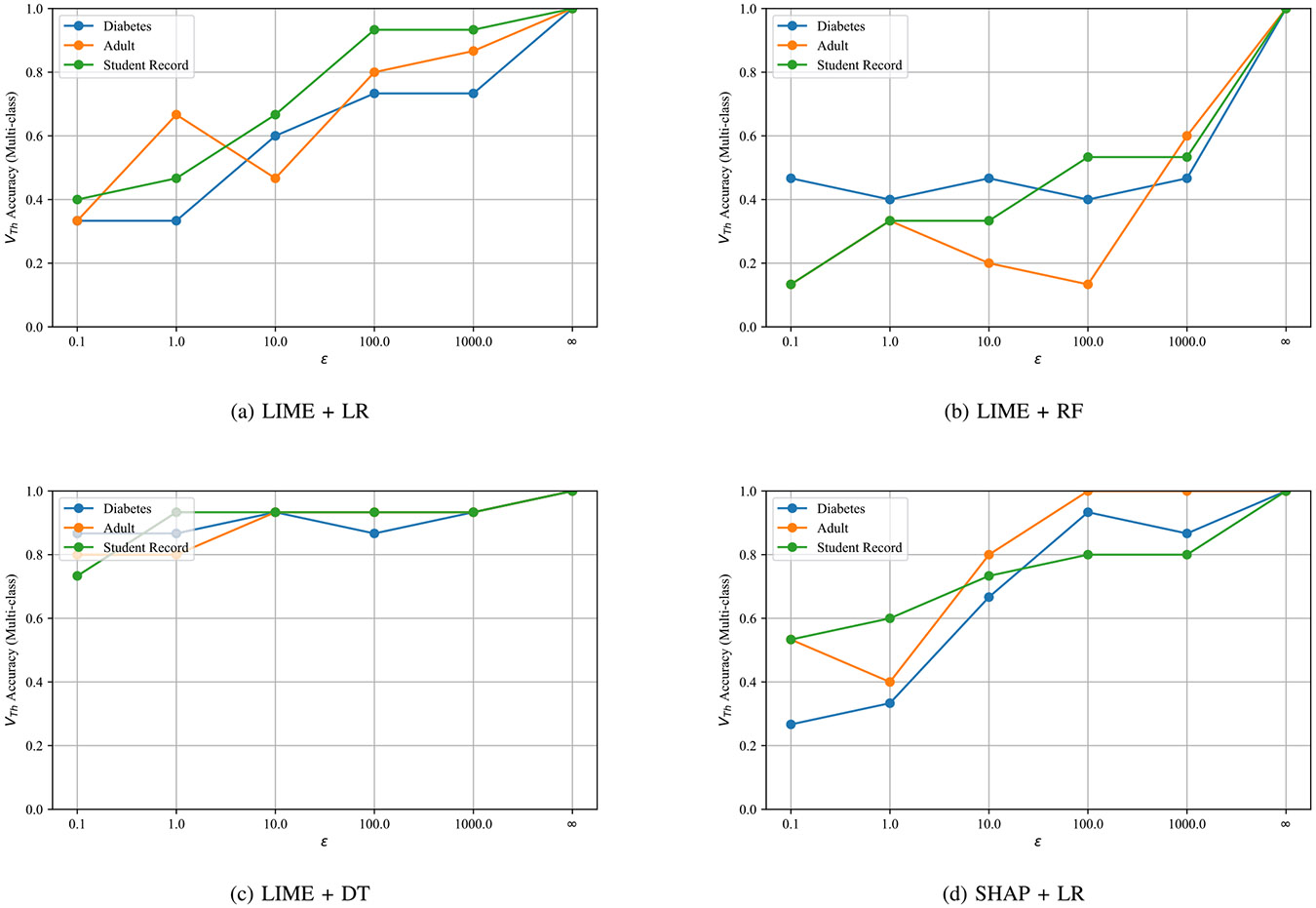
Multi-class verification accuracy with the threshold-based verifier.

For the multi-class task (Fig. 6), the threshold rule is slightly weaker than the ML-based detector, especially at low ε, but remains stable across architectures. With LIME + logistic regression, *Diabetes* grows from ≈ 0.68 at ε=0.1 to 0.79 at high ε; *Adult* and *Student* peak near 0.72 and 0.75. Decision tree and random forest are similar. SHAP + logistic regression performs best, reaching 0.83 on *Diabetes* and > 0.75 on the other datasets at high ε.

### F. INDICATOR ATTRIBUTION STUDY

To quantify the marginal utility of each indicator and assess stability across privacy regimes, we conduct leave-one-out ablation experiments on *Diabetes* (multi-class setting; LIME + logistic-regression detector) at multiple privacy budgets: ε∈{1.0,10.0,100.0,1000.0,∞}.

Starting from the fused vector [ΔA, $D_{\text{KL}}$, ExpDist], we remove one indicator at a time with all other settings fixed. Table 5 presents the complete ablation analysis across the privacy spectrum.

#### F. INTERPRETATION

At ε=∞ (no privacy noise), dropping explanation distance causes the largest degradation (−0.369 F1), confirming that local feature-importance geometry provides the strongest cue under ideal conditions. KL divergence contributes moderately (−0.113), while accuracy shift has minimal impact (−0.045).

Extending the analysis across privacy budgets reveals privacy-dependent indicator importance. At high privacy budgets (ε≥1000), the ranking remains stable: ExpDist > $D_{\text{KL}}>\Delta A$. However, at moderate privacy levels (ε≤100), KL divergence emerges as the most robust indicator, with its removal consistently causing the largest F1 degradation (−0.084 to −0.120). Explanation distance-being computationally expensive to extract and vulnerable to per-feature noise-loses discriminative power at lower ε. At very low privacy budgets (e.g., ε=1.0), all indicators show degraded utility as privacy noise dominates the signal, though the fused baseline still achieves F1=0.262.

This privacy-regime dependence validates our fusion design. While ExpDist excels when privacy noise is minimal (the setting where verification is most accurate), having $D_{\text{KL}}$ and ΔA provides robustness when LIME explanations become unreliable. The fused indicator vector ensures graceful degradation across the privacy spectrum rather than catastrophic failure when any single indicator is compromised. Future work could explore privacy-adaptive weighting that dynamically adjusts indicator contributions based on the operating ε, upweighting $D_{\text{KL}}$ at moderate privacy levels while preserving ExpDist’s dominance at high ε.

### G. IMPACT OF PREPROCESSING ORDER

To investigate whether preprocessing step ordering affects verification accuracy, we conducted experiments with 6 representative orderings selected from the 24 possible permutations of our 4 preprocessing steps (duplicate removal, outlier handling, scaling, and resampling). The selection strategy prioritized diversity: we included orderings achieving the highest and lowest model test accuracy, the standard ordering, and intermediate configurations. All experiments were conducted on the Diabetes dataset with ε=∞ (no privacy noise) to isolate the pure effect of ordering changes. Table 6 presents the results.

#### A) KEY OBSERVATIONS

##### A) ORDER MATTERS MORE FOR VERIFICATION THAN MODEL ACCURACY

Verification accuracy varies by 8.33% (44.87% −53.20% ) across orderings, whereas model accuracy varies only 2.84% (71.40% −74.24% ). This demonstrates that preprocessing order has a stronger impact on verification reliability than on model performance. The 8.33% verification accuracy advantage of the standard ordering over the worst-performing ordering represents a substantial improvement in the framework’s ability to detect preprocessing errors.

##### A) STANDARD ORDERING ACHIEVES OPTIMAL VERIFICATION PERFORMANCE

The conventional preprocessing sequence (Dup → Out → Scl → Res), which follows established ML pipeline design principles, achieves the highest verification accuracy (53.20% ). Notably, orderings that achieved the best model test accuracy (74.24% ) yielded substantially lower verification accuracy (49.47% ). This demonstrates that optimizing preprocessing order for model performance alone does not guarantee optimal verification performance.

### H. RUNTIME

Table 7 lists mean runtimes to train the 15 reference models and extract explanations once (ε=1). Feature dimension, not row count, dominates cost: *Student* (d=36) is slower than the larger *Adult* set.

#### H. RUNTIME COMPOSITION

Coarse timing indicates that over three-quarters of the time is spent in two embarrassingly parallel phases: (i) training the $2^{\sigma }-1$ reference models and (ii) extracting LIME/SHAP explanations. Laplace perturbation of $D_{\text{test}}$ accounts for under 5%, and fitting the final logistic detector takes under one second. End-to-end time therefore scales nearly linearly with additional CPU cores; caching explanations yields further storing.

## VII. DISCUSSION

### A. WHAT DRIVES VERIFICATION PERFORMANCE?

Across datasets and privacy levels, three factors jointly determine accuracy: task granularity, dataset structure, and indicator strength. First, the binary decision (proper vs. improper) is consistently easier than the multi-class diagnosis. The ML detector keeps F1 well above 0.75 even under strong noise (ε=0.1), whereas the multi-class curves in Fig. 5 rise more slowly with ε, especially on *Adult-Income* and *Student-Record*. Second, dataset heterogeneity matters: the relatively homogeneous *Diabetes* table exhibits stable trends, while the other two-richer in categorical attributes and measurement noise-show higher variance. Finally, not all omissions are equally visible: skipping resampling or outlier removal induces larger behavioral drift than failing to drop duplicates, which explains the class-dependent confusions we observe. The ablation in Table 5 clarifies why: removing the explanation-distance feature (*ExpDist*) causes the steepest F1 drop, indicating that local feature-importance geometry carries the strongest signal, with $D_{\text{KL}}$ and the accuracy-shift surrogate contributing additional, complementary information. These effects compound with privacy noise: performance improves monotonically with ε; at ε=0.1 degradation is present but moderate (binary accuracy remains ≫ 0.5), while ε∈[1,100] already yields reliable multi-class diagnosis.

### B. CHOOSING AND DEPLOYING A VERIFIER IN PRACTICE

Two verifier families cover complementary operating points. When the goal is a simple, interpretable gate on model intake (e.g., pass/fail before downstream use), the cosine-threshold rule is attractive: it is training-free, runs fast, and attains > 0.90 accuracy in the binary task even at ε=0.1 (Fig. 6). When finer discrimination is required-small drifts, or identification of the missing step-the ML detector is preferable; it learns a flexible boundary in the fused indicator space and dominates in the binary setting, while performing on par with thresholds in multi-class.

#### Practical knobs and tips:

**FIGURE 7.**
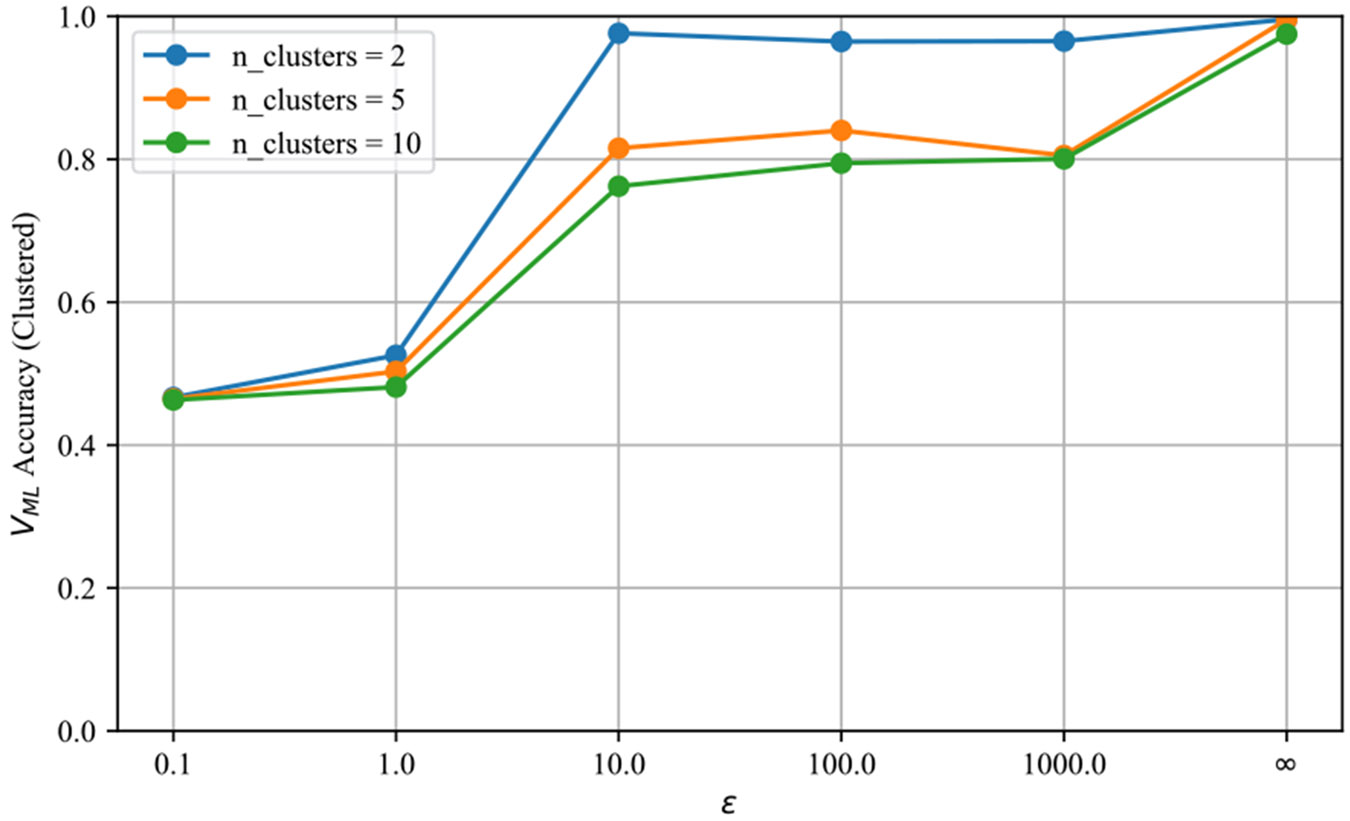
Clustering accuracy from explanation vectors. Fewer clusters (*n* = 2) correspond to the simpler binary decision and yield the highest scores.

(i) *Explainer choice.* SHAP with logistic regression often leads in multi-class accuracy (Fig. 5), but LIME remains competitive and cheaper to compute; either can be used, and both benefit from fusing with $D_{\text{KL}}$ and the agreement surrogate. (ii) *Privacy budget*. Select ε to keep membership-inference power near random guessing (cf. Fig. 4); binary verification remains strong at small ε, while multi-class improves steadily as noise decreases. (iii) *Query budget*. We used 500 queries; more queries generally stabilize explanation statistics, but remember that repeated audits over fresh cohorts should respect the organization’s privacy policy. (iv) *Label-free audits*. When no labeled pipelines are available, clustering only the explanation vectors is a viable fallback: with two clusters, accuracy exceeds 0.9 for ε≥10 and remains > 0.8 at ε=0.1 (Fig. 7). This suggests a practical triage workflow: cluster first to flag likely improper models, then apply the ML detector if finer diagnosis is needed. (v) *Compute*. End-to-end time is dominated by training the reference models and extracting explanations; both are embarrassingly parallel, so wall-clock time scales nearly linearly with CPU cores (Section VI-H).

## VIII. LIMITATIONS AND FUTURE WORK

### Scope beyond tabular data:

Our evaluation is restricted to tabular datasets, where preprocessing steps (e.g., encoding, scaling, resampling) are well-defined and comparable across domains. Extending to other modalities requires addressing two challenges: (i) adapting the privacy mechanism for discrete inputs, and (ii) designing behavioral indicators that reflect modality-specific pipelines.

### Privacy mechanisms for discrete data:

Our current implementation applies the Laplace mechanism (1) to continuous features. For discrete or categorical data-including text tokens-the Laplace mechanism does not apply. Established discrete LDP mechanisms provide equivalent privacy guarantees [53], [54], [55]: Randomized Response (k-RR) outputs the true value with probability $p=e^{\varepsilon }∕(1+e^{\varepsilon })$ and a random alternative otherwise, generalizing to k-ary domains [53]. RAPPOR applies randomized response to Bloom-filter representations for high-cardinality features [54]. For text, discrete LDP would be applied at the token or n-gram level.

### Generalization of behavioral indicators:

The three core indicators-KL divergence, agreement rate, and explanation vectors-generalize naturally to discrete inputs. KL divergence (Equation 4) operates on discrete distributions. Agreement rate is modality-agnostic. LIME and SHAP support discrete features through token-level perturbation and attribution [30], [31]. The verification principle-that preprocessing deviations induce detectable behavioral signatures-extends across modalities. Discrete LDP mechanisms preserve sufficient statistical structure for verification even under strong privacy constraints [55].

### Modality-specific indicator design:

For images, candidates include (1) normalization-statistics drift (per-channel mean/variance), (2) augmentation fingerprints (e.g., flips, crops, color jitter) detected via explanation geometry from Grad-CAM or Integrated Gradients aggregated over superpixels, and (3) KL divergence between calibrated label distributions under test-time augmentations. For text, indicators can track tokenization choices (wordpiece/BPE), lowercasing, stop-word handling, and length normalization by comparing SHAP/LIME attributions on token or phrase spans and measuring distributional shifts in logits across subword boundaries. A practical next step is a small-scale feasibility study on one vision and one NLP benchmark to verify that the fused indicators retain discriminative power when discrete privacy mechanisms replace continuous noise injection.

### Pipeline order and compositionality:

Algorithm 1 assumes a canonical order for optional steps, and Section V-C sketches an enumerate-and-vote extension. In realistic systems, pipelines are better modeled as a DAG with precedence constraints (e.g., encoding before scaling; resampling after splitting). Future work will (1) generate top-k valid topological sorts under those constraints, (2) run the verifier across this candidate set with a budgeted early-stop when confidence concentrates on a small subset, and (3) quantify order sensitivity by injecting controlled reorderings (e.g., scaling ↔ outlier filtering) and measuring the induced indicator deltas. We will also treat non-idempotent steps (e.g., SMOTE) explicitly by fixing random seeds and reporting confidence intervals over multiple resampling draws.

### Indicator attribution and calibration:

While the fused vector [ΔA, $D_{\text{KL}}$, ExpDist] performs well, its components contribute unevenly across datasets and privacy budgets. To reduce redundancy and improve stability at small ε, we plan to (1) learn privacy-aware weights via a sparse meta-learner (e.g., L1-regularized logistic regression) on top of standardized indicators; (2) estimate per-indicator importance with permutation tests and detector-side Shapley values; and (3) attach uncertainty via bootstrap over queries, enabling an abstain decision when confidence is low. This calibration will help operational users tune the accuracy–interpretability–privacy trade-off.

### Adversarial robustness and privacy budgeting:

Our threat model treats the provider as honest-but-fallible; a strategic adversary could attempt to mask missing steps by smoothing logits or manipulating explanations. We will harden the verifier by (1) probing stability under small, randomized input perturbations (disagreement across probes flags tampering), (2) cross-checking explanations from multiple seeds/explainers, and (3) augmenting indicators with simple invariants (e.g., calibration error under temperature scaling). On the privacy side, uniform LDP is conservative; we will investigate data-aware budgets that allocate noise by per-feature sensitivity and attackability, with Rényi-DP accounting to maintain end-to-end guarantees while improving utility.

### Distribution shift and label-free verification:

We evaluated under mild covariate shift. Real deployments face stronger drift (target, conditional, or concept). Future studies will (1) monitor shift via two-sample tests on explanation distributions (e.g., maximum mean discrepancy (MMD) between the distributions of LIME/SHAP explanation vectors [56]) and adapt thresholds accordingly; (2) fine-tune reference models with unsupervised domain adaptation on the noisy queries; and (3) strengthen our label-free variant by combining clustering of explanations with prototype matching and confidence-based self-training. Together, these steps aim to keep verification effective when data populations evolve.

### Computational considerations:

Training up to $2^{\sigma }-1$ reference models and extracting explanations dominates runtime (Section VI-H). We will study (1) caching and reusing explanations across nearby pipelines, (2) early-abandon rules that stop training a reference if indicators already exceed the decision threshold, and (3) lightweight surrogates (e.g., distilled linear probes) to approximate explanations where exact SHAP/LIME is costly. These engineering changes can reduce wall-clock time nearly linearly with cores while preserving accuracy.

## VIII. ETHICAL AND REGULATORY CONSIDERATIONS

All datasets are public and de-identified under their respective licenses (Table 3). Nevertheless, health and education records remain sensitive under the Health Insurance Portability and Accountability Act (HIPAA, United States) and the General Data Protection Regulation (GDPR, EU). Our framework mitigates re-identification risk in two ways: (i) every record released by the researcher is protected by ε-LDP, and (ii) the verifier’s own cohort is perturbed before any query leaves its trust boundary, reducing the feasibility of linkage or membership-inference attacks. The verifier’s output is intended solely for pipeline auditing and not for direct clinical or policy decision-making; downstream users must perform domain-specific validation. No part of this study involves human experimentation or affects individual treatment, and it is therefore exempt from institutional review-board oversight.

## IX. CONCLUSION

This paper presented a privacy-preserving framework that audits whether the declared data preprocessing pipeline was followed when training a machine-learning model. The verifier needs only black-box access to the released model plus a locally differentially private (LDP) dataset, and fuses three complementary behavior indicators-accuracy shift, Kullback–Leibler divergence, and LIME/SHAP explanation vectors.

Across three tabular benchmarks, the machine-learning detector sustained at least 75% F1 in the binary task under a stringent privacy budget (ε=0.1) and performed on par with a threshold rule for multi-class diagnosis. A label-free variant that clusters explanation vectors alone exceeded 90% accuracy once ε≥10, underscoring the discriminative power of explanations.

These results show that model behavior indicators can expose subtle preprocessing omissions while respecting strong local differential privacy. Future work will extend the verifier to modality-specific pipelines (vision and text), investigate adaptive indicator weighting via attribution analysis, and develop semi-supervised detectors that remain effective under severe distribution shift.

## Figures and Tables

**TABLE 1.** Frequently Used Symbols and Notation

Symbol	Description
D	Original training dataset
$D_{\varepsilon }$	LDP-protected version of D
$D_{\varepsilon }^{\prime }$	LDP data after improper preprocessing
$D_{\text{test}}$	Verifier’s private query set
$M_{R}$	Model released by the researcher
$M_{\varepsilon }$	Verifier’s reference model on $D_{\varepsilon }$
$M_{\varepsilon }^{\prime }$	Model trained on $D_{\varepsilon }^{\prime }$
E	Explanation vector (LIME/SHAP); $E^{x}$ for sample x
A	*Label-free agreement rate* on $D_{\text{test}}$ (accuracy surrogate; no ground-truth labels)
$D_{\text{KL}}$	KL divergence between two output distributions
O	Behavior tuple (E, A, $D_{\text{KL}}$)
$V_{\text{ML}}\;∕\;V_{T}$	ML-based / threshold-based verifier
ε	LDP privacy budget

**TABLE 2.** Threats Considered and Built-in Mitigations

Actor	Potential attack / failure	Mitigation in framework	Residual risk
Researcher (honest but fallible)	Accidentally omits one or more preprocessing steps	Behavior indicators [ΔA, $D_{\text{KL}}$, ExpDist] flag drift; Algorithm 1 returns missing-step set	False-negative risk if behavioral drift ≤ τ
Verifier	Membership inference on $D_{\varepsilon }$ or on noisy queries	Local DP (Laplace noise) applied to both $D_{\varepsilon }$ and $D_{\text{test}}$ empirical attack power ≤ 0.5 for ε≤100 (Fig. 4)	Bounded by chosen ε

**TABLE 3.** Dataset Characteristics. Num. = Numeric, Cat. = Categorical

Dataset	Rows	#Feat.	Num./Cat.	Minority ratio
Diabetes	253,680	21	7 / 14	13.9% (diabetes)
Adult	48,842	14	6 / 8	24.0% (≥ $ 50k)
Student	4,424	36	11 / 25	22.7% (drop-out)

**TABLE 4.** Complete Preprocessing Case Definitions. Steps: Dup=duplicate Removal, Out=outlier Handling, Scl=scaling, Res=resampling. All Cases Include Two Mandatory Steps (Drop Missing Values, Encode Categorical) That Always Execute First.

Case	Steps Included	Steps Missing	Category
0	[Dup, Out, Scl, Res]	None	Proper
1	[Dup, Out, Scl]	Res	1-step
2	[Dup, Out, Res]	Scl	1-step
3	[Dup, Scl, Res]	Out	1-step
4	[Out, Scl, Res]	Dup	1-step
5	[Dup, Out]	Scl, Res	2-step
6	[Dup, Scl]	Out, Res	2-step
7	[Dup, Res]	Out, Scl	2-step
8	[Out, Scl]	Dup, Res	2-step
9	[Out, Res]	Dup, Scl	2-step
10	[Scl, Res]	Dup, Out	2-step
11	[Dup]	Out, Scl, Res	3-step
12	[Out]	Dup, Scl, Res	3-step
13	[Scl]	Dup, Out, Res	3-step
14	[Res]	Dup, Out, Scl	3-step

**TABLE 5.** Ablation Study Across Privacy Budgets (Diabetes, Multi-Class Task, LIME + Logistic Regression). Indicator Importance Shifts With Privacy Regime.

Feature Set	ε=1	ε=10	ε=100	ε=1000	ε=∞
All three	0.262	0.306	0.365	0.586	**0.762**
w/o ΔA	0.246	0.304	0.365	0.572	0.717
	−0.016	−0.002	+0.000	−0.014	−0.045
w/o $D_{\text{KL}}$	0.178	0.186	0.249	0.495	0.649
	−0.084	−0.120	−0.116	−0.091	−0.113
w/o ExpDist	0.489	0.304	0.393	0.393	0.393
	+0.227	−0.002	+0.028	−0.193	−0.369

**TABLE 6.** Impact of Preprocessing Order on Verification Accuracy. Results on Diabetes Dataset With ε=∞ (No Noise). Verification Accuracy: Multi-Class Classifier Accuracy for Identifying Preprocessing Cases (0–14). Model Accuracy: Test Set Performance for Proper Preprocessing (Case 0). Dup=duplicate Removal, Out=outlier Handling, Scl=scaling, Res=resampling.

Ordering Type	Steps	Model Acc.	Verif. Acc.
Standard	Dup→Out→-Scl→Res	73.33%	53.20%
Alternative 1	Scl→-Res→Dup→Out	72.52%	52.00%
Alternative 2	Scl→Dup→Res→Out	74.24%	49.47%
Alternative 3	Dup→Scl→Res→Out	74.24%	49.13%
Alternative 4	Out→Scl→Res→Dup	71.40%	45.73%
Alternative 5	Scl→Out→Res→Dup	71.44%	44.87%
Range	–	2.84%	8.33%

**TABLE 7.** Mean Runtime Per Run (seconds)

Dataset	Rows	Time (s)
Diabetes	253,680	652
Adult-Income	48,842	442
Student-Record	4,424	753

## Box 1 - Running Example

### Setting.

A data provider shares an income-prediction model trained on the Adult Income table.

#### Pipeline the provider claims to have used

1. remove duplicate rows
2. convert categorical columns to one-hot vectors
3. scale numeric columns to zero mean and unit variance

### Hidden slip-up.

Step 3 (scaling) failed silently, so the true “missing-step set” is S={scaling}.

#### What the verifier can do

- Holds 500 private test rows, adds Laplace noise for ε-local differential privacy, and queries the released model (call it $M_{R}$).
- Trains three reference models on the same noisy rows: one with every step correct, one with scaling omitted, and one with both one-hot encoding and scaling omitted.

### How the verification works

For each reference model the verifier compares its behavior with $M_{R}$: overall accuracy shift, KL divergence between prediction probabilities, and distance between LIME/SHAP explanations. These three numbers form a feature vector; a small logistic classifier chooses the reference that looks most similar to $M_{R}$.

### Result.

The “no-scaling” reference scores highest, so the verifier outputs $\hat{S}=\{\text{scaling}\}$ and flags the provider’s oversight.

### Scenario.

A hospital’s analytics team (researcher) trains a logistic regression model to predict 30-day readmission risk from electronic health record (EHR) tables. The declared preprocessing pipeline is drop-duplicates → one-hot-encode →scale-numeric.

### Inadvertent error.

During an automated retraining cycle, the scaling step crashes silently; the model $M_{R}$ is therefore fitted on unscaled lab values. Because numeric features dominate the loss, coefficients are inflated yet the ROC–AUC on the internal test set remains 0.83, giving no obvious warning.

### Verification set-up.

Before deploying the model, an insurance provider (verifier) evaluates it:

1. 500 anonymized patient visits form $D_{\text{test}}$.
2. Each record is perturbed with Laplace noise, yielding $D_{\varepsilon }$ that satisfies ε-LDP.
3. Reference models $M_{\emptyset }$ (proper pipeline) and $M_{\left\{\text{SCALE}\right\}}$ (scaling omitted) are trained on $D_{\varepsilon }$.
4. For every visit the verifier collects E (SHAP values), ΔA, and $D_{\text{KL}}$ as in Table 1.

### Outcome.

Algorithm 1 assigns the highest probability to the missing-step set {SCALE}, flagging the model before any policy decisions rely on it.

## Footnotes

1 https://www.kaggle.com/datasets/ - “CDC Diabetes Health Indicators”.
